# Impact of biological education and gender on students’ connection to nature and relational values

**DOI:** 10.1371/journal.pone.0242004

**Published:** 2020-11-05

**Authors:** Matthias Winfried Kleespies, Paul Wilhelm Dierkes

**Affiliations:** Bioscience Education and Zoo Biology, Goethe-University, Frankfurt, Germany; University of Leeds, UNITED KINGDOM

## Abstract

The new concept of relational values (RVs) is gaining more and more attention in environmental research, but empirical analyses are still rare. However, this type of research is necessary because the RVs have an influence on environmental behavior. To evaluate the impact of biological education on attributing higher importance to RVs and connectedness to nature, we compared the connection to nature scores (using the inclusion of nature scale (INS) and connectedness to nature scale (CNS)) and RV scores of biologically interested high school students (n = 417) with first year (n = 593) and advanced biology (n = 223) students. While high school students showed significant lower connection to nature scores than university students, there was no significant difference in RVs between the test groups. These results suggest that there is a lack of factors in the university study of biology that can change RVs. The gender comparison of RVs and connection to nature showed a significant higher RV score for females while INS and CNS did not show a gender difference. Thus, the study makes an important contribution to the research, as it was able to prove that gender has an influence on a person's RVs but not on their connection to nature.

## Introduction

In our modern Western world, the relationship of humans to nature is undergoing a massive change. The digitization of our society and alienation from nature are becoming increasingly problematic in both in professional and private life. Adults and children spend more time indoors and less time in a natural environment. Robinson and Silvers [1] used a one-day diary to show that 51% of American adults spend nearly no time outside. A national report revealed that the increasing time Americans spend with electronic media and devices and greater isolation from nature are major causes of their growing disconnection from nature [2]. Many recent studies have reported a significant proportion of time that children and adolescents spend with media. In a survey conducted among Spanish secondary school students, half of the participants reported watching television for more than two hours on a normal weekday. Additionally, the number of students surveyed using a computer for more than two hours a day was over 60% [3]. The Youth Report 2016 surveyed 1,253 sixth- and ninth-graders in North Rhine-Westphalia (Germany) and found that more than half (57%) of the participants stated that they spend more than three hours a day at a screen (e.g., TV, smartphone, tablet; [4]). As a result of this development, the bond between people, and especially children, and the environment decreases. The split of humans and nature is one of the potential explanations of the growing environmental problems [5].

A more pronounced human-nature connection has the potential to increase conservation performance [6]. People with higher connection to nature are more motivated to show environmentally friendly behavior [7–11]. In addition, connection to nature reveals a positive correlation with environmentally reasonable behavior [12] and it appears to be a strong predictor for pro-environmental behavior [8, 13, 14]. This is why a reconnection of people and nature is demanded [15].

**Fig 1:**
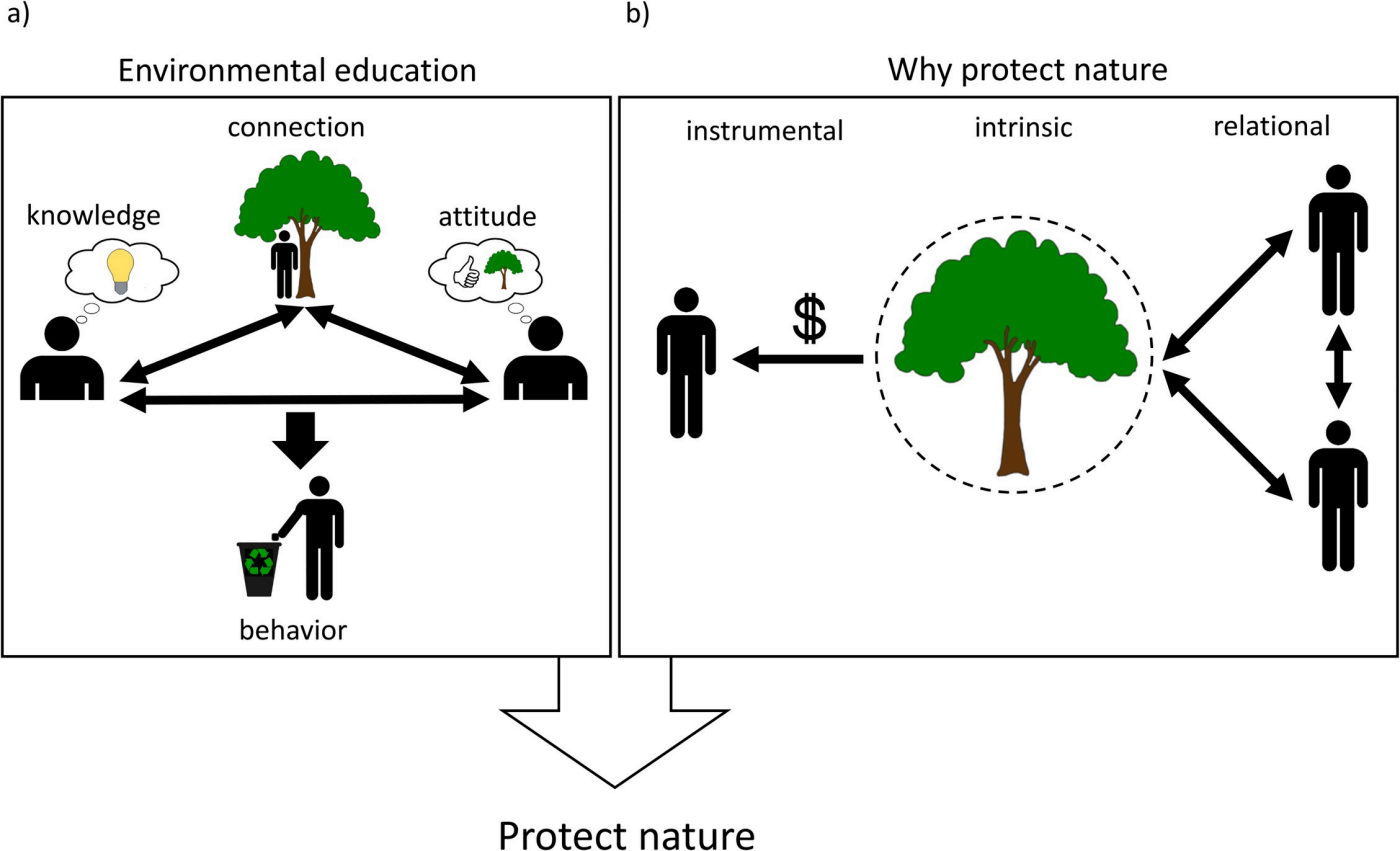
a) Environmental education incorporates the combination of knowledge, attitude and nature connectedness to reach environmental behavior to protect nature. b) The protection of nature is justified in environmental literature with instrumental, intrinsic and relational arguments.

For this reason, current environmental education research focuses not only on expanding environmental knowledge [16, 17] or changing environmental attitudes [18, 19], but especially on increasing the connection to nature ([20–22]; Fig 1). However, the reasons why people should protect parts of nature at all are promoted in only a few environmental education programs [23].

This is wasted potential, because these reasons are important initiators for environmentally responsible behavior [24]. So what is the motivation for humans to protect nature or in other word: Why do people protect nature?

The question of why we should care for nature and make efforts to preserve it is answered in different ways. Conservationists and philosophers argue with the intrinsic values of nature. The intrinsic values are a concept that gives nature a value of its own even when humans do not benefit directly or indirectly from it [25]. Biodiversity and the different species are considered to have value of their own that cannot be denied or recant [26]. These intrinsic values can be understood in two different ways: As subjective and objective intrinsic values [27]. In the subjective view, intrinsic values are created by the evaluation or judgment of an observer and in the objective view the intrinsic values of nature are independent of the judge of humans. Both perspectives of intrinsic values are considered to be crucial for conservation biology [28], although there are doubts concerning the ability of intrinsic values to guide people to conservation decisions (e.g. [29]). For this reason, a different type of value is often used in economics, namely instrumental values or a utilization view of nature. Nature is not valued for its own sake but for the services and benefits it provides for humans [30] and thus the value of nature is determined solely by the measurable, anthropocentric value it offers. Costanza et al. [31] calculated the value of ecosystem services that nature provided for the year 2011 to 125 trillion dollars what is in total nearly the double of the global gross domestic product in the same year [32]. The supporters of the instrumental view argue that instrumental arguments are a powerful tool for a wider range of people [33], but these arguments are criticized for selling nature out or rather reduce the value of nature [34]. Also the opportunity to conserve things that have little or no instrumental value for humans is missing [33].

### Relational values

Because humans decisions concerning nature are not only based on the exploitability or the intrinsic value of nature, an additional kind of values was recently introduced: relational values (RVs) [35–37]. People also consider what they believe is the right and appropriate way to deal with nature. This also includes what they think is conducive to live a satisfying and meaningful life. RVs incorporate relationships people have to nature but also relationships between people that involve nature. So RVs have a mainly personal component and one that involves the human collective. In contrast to intrinsic values RVs are not exclusively assigned to objects but develop out of the relationship to them [35].

The description of the RV concept shows that RVs do not stand for themselves, but overlap with other prominent value concepts. The RVs contain a certain amount of assigned values (values of objects) in the form of eudaimonic values. Furthermore, moral values (what is right and what is wrong) are part of the RV concept [38]. However, RVs can be distinguished from the concept of value as it is often used in environmental or social psychology. There, values are often defined as goals that serve as a guideline in the life of a person and thus have an influence on behavior, attitudes and beliefs [39]. This concept of values as goals or also called held values differs from RVs, because held values refer to abstract characteristics or general ideals (like fairness or courage), while RVs refer to an object [38]. In the context of nature, Arias-Arévalo et al. [40] define the concept of value as reasons why certain parts of nature are important to an individual or social group. This definition can be easily applied to the RV concept. Thus, values can have a direct influence on behavior, but they can also influence behavior via mediators such as beliefs attitudes and norms [39].

Introducing RVs gained attention and the concept was applied in different research areas [38]. Due to the novelty of the RV concept, only some empirical studies are published. Most RVs research focuses on theoretical framework (e.g. [38, 41–43]) or qualitative methods [40, 44], whereas quantitative research is conducted only in a few publications [40, 45–48]. However, quantitative studies are very important to bring empirical evidence on the topic of RVs and to prove hypothesizes and assumptions theoretical analysis and qualitative research set. Approaching quantitative data also offers the opportunity to identify core RVs in different cultures and support communication and cooperation on this way. Thirdly, a quantitative recording of RVs gives politicians the possibility to make decisions based on public views [43].

The construct of RVs can be classified into the complex concept of human nature relationships that is applied in a number of research fields [49]. In a multidisciplinary review of research papers on human-nature connections (HNC) from 1984 onwards, Ives et al. [50] found that the topic is becoming increasingly important. Despite the heterogeneity and the large number of publications, their cluster analysis showed that the publications on the HNC can be divided into three subgroups: HNC as mind, as experience or as place. Publications from the mind cluster deal with the cognitive aspects of the HNC of an individual. Often this view is used in psychology and nature is not specified more precisely. Furthermore, this connection is often used to explain an effect on environmental behavior. HNC as experience capture the experience with a location, while HNC as place reflect the emotional and relational interaction between people, special nature places and landscapes [50]. The RV concept, as used in this study, includes parts of all three clusters of HNC. Thus the RVs include the relationship between people, places and nature [35] as in HCN as place and HCN as experience, but also elements of the emotional connection of nature [47, 51] as in HNC as mind. Another kind of HNC from environmental psychology and environmental education research that can be assigned to the HNC as mind is the concept of connection to nature [13, 52]. In this cluster the concept of nature is often not defined in detail, psychometric scales are used as quantitative measurement methods and a special focus is put on the cognitive connection to nature [50].

### RVs and connection to nature

In recent years especially environmental education programs focus on promoting connection to nature. Although connection to nature is now a commonly used construct, there is no universal definition. Clayton [7] emphasizes the role of personal identity in the connection to nature, while other authors focus more on the emotional component of being connected to nature [8, 13]. For this reason, a number of different measuring instruments have been developed in research. For example the connectedness to nature scale (CNS) by Mayer & Frantz [13] or the inclusion of nature in self scale (INS) by Schultz [52].

Schultz [52] distinguishes three dimensions in his construct of the "inclusion of nature in self", which together form the concept of connection to nature: The cognitive dimension describes how strongly a person sees himself as part of nature. The affective dimension indicates whether a person cares about nature. The behavioral dimension deals with the question whether a person is motivated to act in the best interest of nature. The cognitive level is the starting point and forms the basis for the affective level, which in turn is the prerequisite for the behavioral level. Although the various constructs for measuring the connection to nature differ, it has been shown that they are all related and probably measure a common construct [53, 54].

Despite the fact that connection to nature and RVs describe HNC, there are some similarities, but also differences. The first commonality is, as mentioned, that both concepts are HNC. In typifying human-nature relations in the form of "human-nature relational models", Muradian & Pascual [42] present seven elementary models that can be distinguished by conceptual categorization. Both RVs and connection to nature can be assigned to the same human-nature relational model (stewardship). But also the connection to nature concept can be counted as part of the stewardship model, since humans are seen as part of nature, which is the basic idea of the connection to nature [52]. The second commonality is that in both concepts the personal relationship to nature plays an important role. Connection to nature describers the emotional [13, 55] and cognitive [56] connection of the individual to nature. This is also a part of the RV concept [47, 51]. The third commonality is that both concepts have an influence in the environmental behavior of a person (e.g. [8, 13, 14, 55, 57]). Despite these similarities, there are also some differences between the concepts. The first difference is that the RVs also include other aspects which go beyond the construct of connection to nature. For example, community, care, identity and kinship are part of the RVs [35, 45, 47, 51] but not of connection to nature. The second difference is that while the connection to nature concept deals with the connection of an individual to nature [13, 52, 55], the RVs also refer to concrete values what people see as important and meaningful about nature in relation to other people [35].

Numerous studies have shown that environmental education is an effective way to increase the connection to nature [21, 22, 58, 59]. Studies by Britto dos Santos and Gould [51] and Uehara et al. [46] suggest that RVs are dynamic and can potentially be strengthened by environmental education. In this context, this study will examine whether intensive environmental education, e.g. biology studies, has an impact on RVs and connection to nature. For this purpose, the RVs and the connection to nature of biologically interested high school students, biology students in their first year of study (referred to in the text as "first year students") and biology students in higher semesters (referred to in the text as "advanced students") will be compared.

A factor that has a special influence on a number of environmental variables is gender. In several studies, a more positive attitude towards the environment has been observed among women [16, 60, 61]. Men tend to see nature as something that should be used to their own advantage [62, 63], while women see nature as something worth preserving [64]. It was also observed that women have a higher level of environmental knowledge [65], are more concerned about the environment [66] and tend to be more willing to protect nature [67]. For the concept of connection to nature there are contradictory results. While some studies have found no difference between men and women [13, 68], there is also evidence for a higher connection to nature among women [69]. This study will also investigate whether there is a difference between the genders in their connection to nature. For the RVs, there are only few studies on potential gender effects (e.g. [48, 70]). Therefore, this study is intended to provide important evidence about the impact of gender on the RVs.

## Methods

### Measurement

#### Measuring RVs

Arias-Arévalo et al. [40] used an open-ended questionnaire to identify environmental values (defined as reasons why parts of nature are important for people or a social group) concerning the Otún River watershed. The study points out that RVs and intrinsic values are frequently mentioned, while only a minority of the participants brought up instrumental values. People living in rural areas showed higher relational and intrinsic values than people living in urban areas. Chapman [44] conducted interviews with 22 farmers and land managers and identified RVs as crucial key values. Based on the RVs explanations by Chan et al. ([35], Fig 1), Uehara et al. [46] developed 7 RVs statements customized to the Hinase district in Japan to measure RVs. With this quantitative approach, they showed the importance of RVs in the Hinase district for people living there. A further empirical instrument was developed by Klain et al. [45]. They adapted 7 value statements on RVs from studies of cultural ecosystem service to survey participants on an online survey, Costa Rica farmers and tourist. All test groups showed high RV scores (a mean score of 4 on a 5-point Likert scale). Additionally, Klain et al. [45] showed that RVs and the *New Ecological Paradigm (NEP)* by Dunlap et al. [9] are distinct constructs.

The questionnaire used by Klain et al. [45] and Uehara et al. [46] provided meaningful results for their survey group. For our study we applied the seven items of Klain et al. [45] because the questions show a good fit with the concept of RVs and the reliability and validity of the instrument are proven [47]. Because the questionnaire of Klain et al. [45] was used to survey farmers and tourist, we changed the wording of one item from “How I manage the land […]” to “How we manage the land […]”. The statements had to be rated on a 5-point Likert scale.

#### INS

The inclusion of nature in self-scale (INS) is an instrument used for measuring connection to nature. The scale was developed by Schultz [52], who modified the “Inclusion of Other in the Self Scale” developed by Aron et al. [71]. INS is a graphic single-item construct (S1 Fig). It consists of seven circle pairs (1 to 7) labeled as “nature” and “me.” The pairs differ in their degree of overlap, from completely separated from each other (separated from nature) to completely overlapping (very strongly connected to nature). The INS has been used frequently and has shown meaningful results (e.g., [21, 22, 58, 72, 73]).

#### CNS

The connectedness to nature scale (CNS) was developed by Mayer & Frantz [13] to measure people’s affective connection to nature. While other measurement instruments for example the New Ecological Paradigm by Dunlap [9] measures cognitive beliefs the focus of the CNS is on the emotional connection to nature [13]. Perrin & Benassi [74] criticized the CNS for missing the aim to measure the emotional connection to nature but agreed that the CNS is a tool to quantify connection to nature. The CNS has shown high correlation with other frequently used connection to nature measurements [53, 54] and its reliability is proven frequently (e.g., [75–78, 69, 79]). Because of the limited time during the survey and to keep the questionnaire compact we used the seven highest loading CNS items provided by Mayer & Frantz [13]. As with the RVs test instrument, a 5-point Likert scale was used.

### Participants

In total, the sample consisted of 1233 participants (64.7% female, 33.8% male, 1.5% no answer). 816 participants (age_mean_ = 20.26) were students from the Goethe-University in Frankfurt (Germany) and 417 were high school students from local schools. Most of the students (n = 593) were first year undergraduate students in biology or biology teacher training while the remaining 223 participants were advanced students in biology or biology teacher training. The first year university students were surveyed during a weekly internship every biology student and biology teacher trainee has to take in the first two semesters of studying at the Goethe-University Frankfurt. The advanced students completed the questionnaires during seminars for higher semester in the department of Bioscience Education and Zoo Biology. Participation in our survey was voluntary and the students were allowed to hand in empty questionnaires, so no one was forced to fill out the survey. The data collection took place in winter semester 2018/19.

The high school students (age_mean_ = 17.51) attended in out-of-school education programs in the department of Bioscience Education and Zoo Biology. The majority of the high school students (98.6%) had chosen biology as a teaching subject (76.0% as major and 22.6% as basic course; the remaining 1.4% did not have biology at school). Before the regular program started, the students had to fill out the questionnaires. For the participation, the school groups received a reduced price for the program or benefits such as a free guided zoo tour. If individual students did not participate in the survey, they did not have any disadvantage and still received the reward. The students and their parents were informed beforehand in writing about the survey and that, participation is voluntary and that there is no disadvantage if the questionnaire is not completed. Participants under the legal had to bring a signed letter of agreement by their parents. Privacy policy has been respected. The data were obtained between July 2018 and May 2019.

### Analysis

All statistical analyses were executed using IBM SPSS 24. To confirm the single factor solution of the reduced CNS a principal component analysis (PCA) with varimax rotation was executed, after the Barlett-test and the Kaiser-Meyer-Olkin-test were applied. Similar to the original study by Klain et al. [45] the RVs items were forced to a single factor in a PCA after the Barlett-test and the Kaiser-Meyer-Olkin-test approved sampling adequacy. The Mann-Whitney-U test was applied to examine the difference between genders for RVs, INS and CNS after the Kolmogorov-Smirnov-test did not confirm normal distribution. For the comparison of high school, first year and advanced students the Kruskal-Wallis-test was used and for significant results a post-hoc test (Dunn-Bonferroni-test) applied. To evaluate the differences for significant results the effect size was calculated using the formula r = $\frac{z}{\sqrt{N}}$ that is suggested by Fritz et al. [80] for nonparametric data. For a comparability with other studies we converted the r in d with the formula d = $\frac{2r}{\sqrt{1-r^{2}}}$ by Borenstein et al. [81]. The correlation between RVs, INS and CNS were determined using Pearson correlation coefficient.

## Results

After the Barlett-test was highly significant (p < .001) and the Kaiser-Meyer-Olkin-test confirmed the adequacy of sampling (KMO = .855), the first PCA with orthogonal rotation was used to confirm the one factor solution of the CNS. When the lowest loading item (< .4) was removed the remaining six CNS items showed high factor loadings (> .6) on a single factor accounting for 54.69% of the variance (Table 1).

**Table 1:** Result of the principal component analysis with orthogonal rotation for the six CNS items (Mayer & Frantz, 2004).

		MEAN ± S.D.	FACTOR LOADING
**CNS_1**	I often feel part of the web of life.	3.04 ± 1.11	.828
**CNS_2**	Like a tree can be part of a forest, I feel embedded within the broader natural world.	2.78 ± 1.16	.793
**CNS_3**	I often feel a sense of oneness with the natural world around me.	2.82 ± 1.08	.765
**CNS_4**	I think of the natural world as a community to which I belong.	3.25 ± 1.12	.764
**CNS_5**	When I think of my life, I imagine myself to be part of a larger cyclical process of living.	3.38 ± 1.20	.642
**CNS_6**	I feel as though I belong to the Earth as equally as it belongs to me.	3.08 ± 1.23	.621
	*α* = .829		

Note. S.D. = Standard Deviation; the mean value of the whole scale is 3.06 ± 0.84

The six applied CNS items show high factor loadings and a good Cronbach alpha (.82) reaching a similar alpha score as Mayer & Frantz [13] in the original study (.84) and Pasca et al. [82] for a reduced CNS scale (.866). The items show a clear one factor solution and the data confirm the internal consistency and the structure of the well-known CNS scale for our translated version of the measurement tool and our sampling group, confirming the shorted version as a usable measurement tool.

The second PCA was applied to verify the one factor solution for the seven RV items developed by Klain et al. [45]. The requirements for a PCA were meet (Barlett-test p < .001; KMO = .785) and the forced single factor solution showed acceptable factor loadings (> .5) and Cronbach’s alpha (.774). The first factor accounted for 40.16% of the variance (Table 2).

**Table 2:** Result of the principal component analysis with oblique rotation for the seven RV items (RV scale from Klain et al. 2017).

		MEAN ± S.D.	FACTOR LOADING
**RV_IDEN**	I have strong feelings about nature (including all plants, animals, the land, etc.) these views are part of who I am and how I live my life.	3.21 ± 1.19	.744
**RV_ RESP**	How ***we*** manage the land, both for plants and animals and for future people, reflects my sense of responsibility to, and so stewardship of the land.	4.14 ± 0.99	.671
**RV_WILD**	I often think of some wild places whose fate I care about and strive to protect, even though I may never see them myself.	3.36 ± 1.28	.659
**RV_KIN**	Plants and animals, as part of the interdependent web of life, are like “kin” or family to me, so how we treat them matters.	3.21 ± 1.23	.645
**RV_HEALTH**	My health or the health of my family is related one way or another to the natural environment.	3.84 ± 1.07	.625
**RV_OTHER**	Humans have a responsibility to account for our own impacts to the environment because they can harm other people.	4.46 ± 0.84	.543
**RV_COMM**	There are landscapes that say something about who we are as a community, a people.	3.53 ± 1.21	.520
*α* = .744			

Note. S.D. = Standard Deviation; the mean value of the whole scale is 3.68 ± 0.71

The seven RV items show acceptable factor loadings and Cronbach alpha (.744). Using only six of their seven items Klain et al. [45] reached an alpha score of .80. Even when the RV items do not show a perfect fit with the forced single factor solution it can be assumed that tested measurement scale is an adequate measurement tool for RVs.

**Fig 2:**
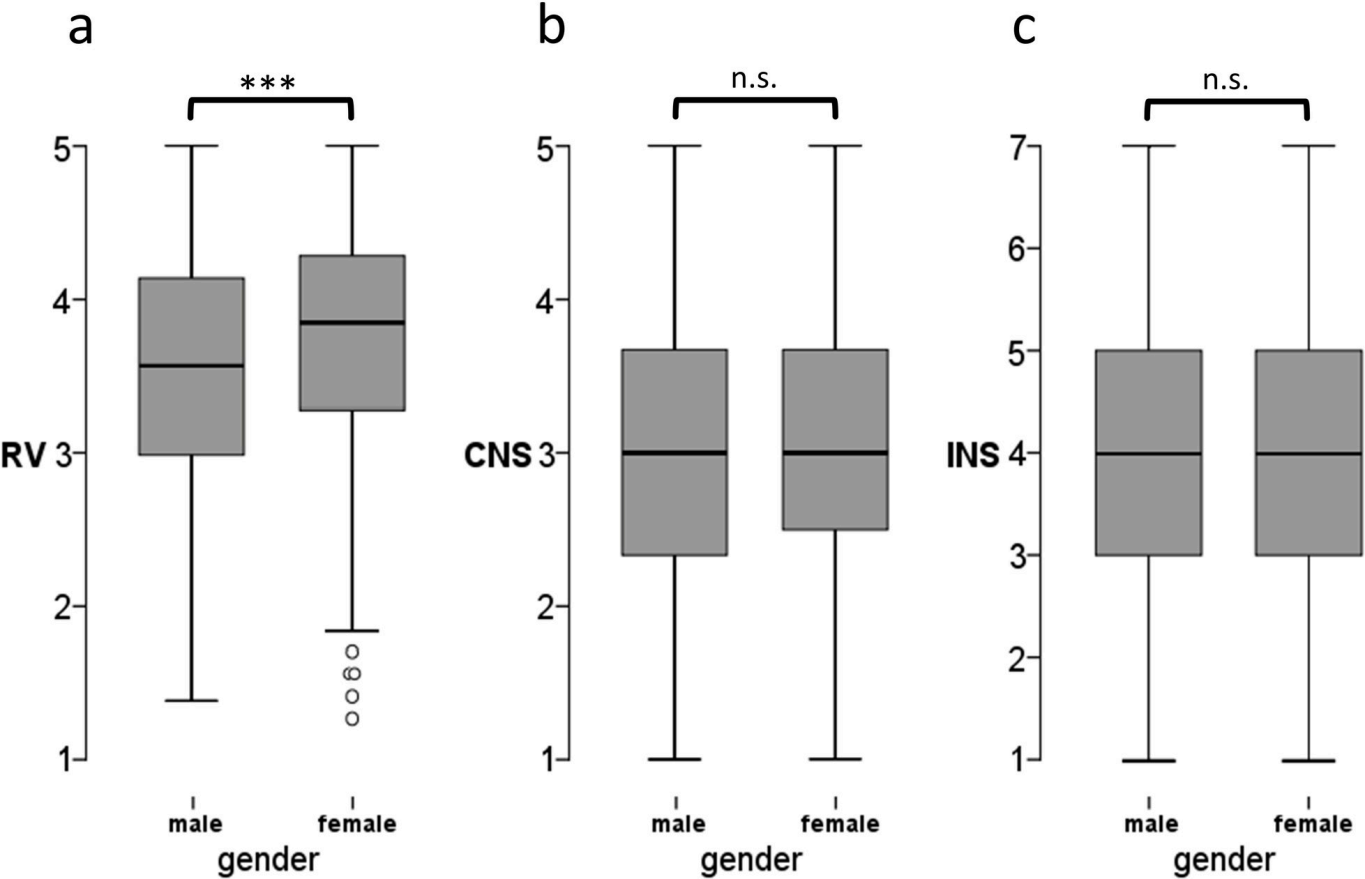
Boxplots comparing a) RVs, b) CNS and c) INS by gender. Significant shifts are marked with * p < 0.05, ** p < 0.01, *** p < 0.001 (n.s. = not significant).

The Mann-Whitney-U test showed a significant result for the gender comparison of RVs (p < .001; Mean_male_ = 3.55; S.D. = .70; Mean_female_ = 3.75; S.D. = .70) with an effect size of d = .273. For the INS (p = .507; Mean_male_ = 4.16; S.D. = 1.35; Mean_female_ = 4.19; S.D. = 1.15) and CNS (p = .398; Mean_male_ = 3.02; S.D. = .90; Mean_female_ = 3.06; S.D. = .81) the Mann-Whitney-U did not show a significant gender difference (Fig 2). For RVs, the Kruskal-Wallis-test showed no significant result (p = .328) indicating no group differences between high school, first year and advanced students.

**Fig 3:**
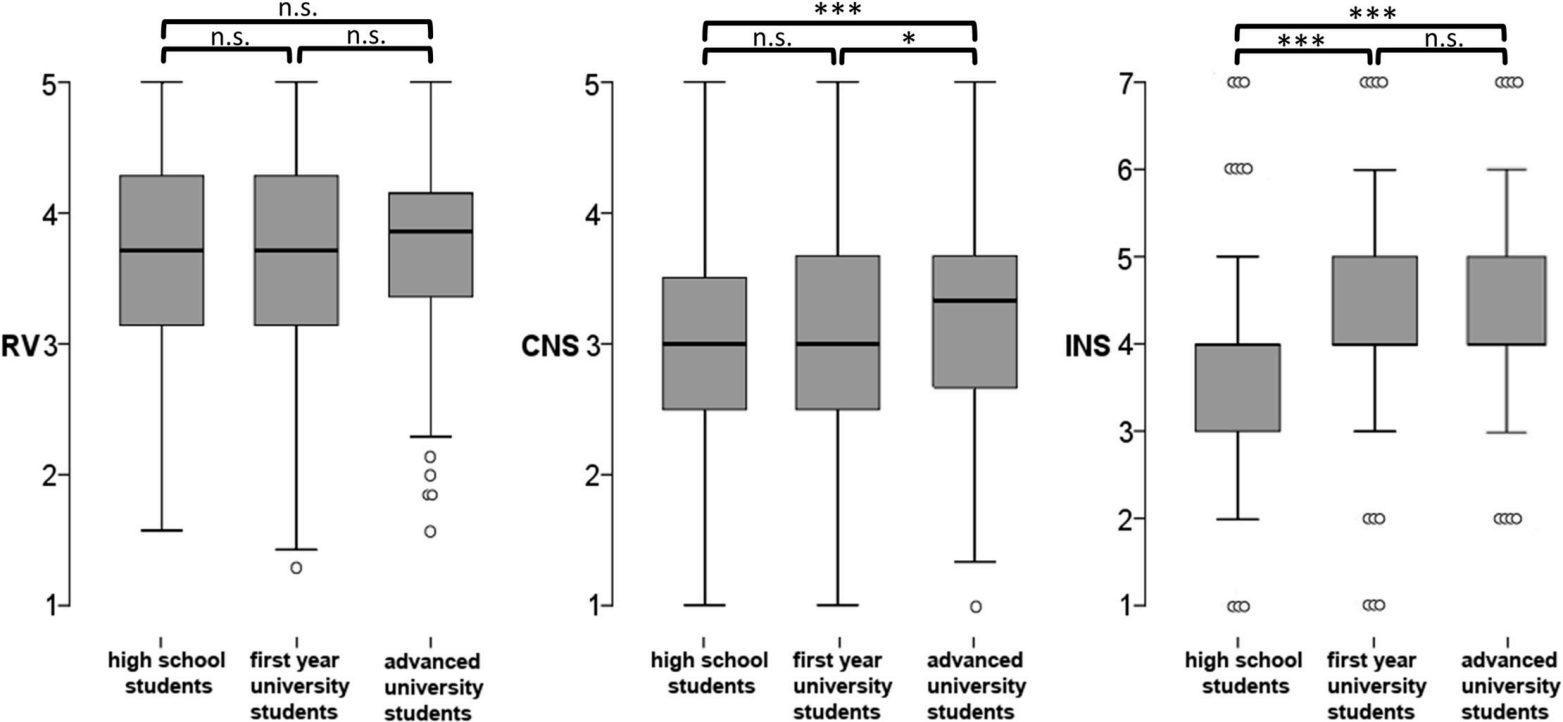
Boxplots of the CNS, INS, and RVs grouped in education levels. INS shows a significant difference between high school and first year students and between high school and advanced students. The CNS shows differences between high school and advanced students and between first year and advanced students. However, RVs show no differences between the three groups. Significant shifts are marked with * p < 0.05, ** p < 0.01, *** p < 0.001 (n.s. = not significant).

The Kruskal-Wallis test for the CNS was significant (p < .001) as well as the pairwise comparison of high school and advanced students (p < .001) with an effect size of d = .328. The difference of high school and first year students was not significant (p = .137), while the comparison of first year students and advanced students showed a significant result (p = .023) with a small effect size (d = .188). The Kruskal-Wallis-test for INS was highly significant (p < .001) indicating a group difference. The post-hoc test revealed significant (p < .001) results between high school and advanced students with an effect size of d = .360. High school and first year students showed a significant difference as well (p < .001) with a slightly higher effect size (d = .378). For the comparison of first year students and advanced students the test showed no significant difference (p = .932;) Fig 3). The mean values and standard deviation for each item of the RV scale, CNS and INS categorized by gender and education level can be found in S1 Table.

A correlation of r = .567 (p < 0.001) could be determined between CNS and INS. Between RVs and the INS the correlation was r = .438 (p < 0.001) and between RVs and CNS r = .533 (p < 0.001).

## Discussion

Compared to other empirical studies, our test group achieved lower scores for the RVs. Klain et al. [45] were able to determine a mean value of 4.0 ± 0.68 for the RVs when using the same test instrument, whereas in this study a mean value of 3.68 ± 0.71 was achieved. A possible explanation for the difference could be the composition of the test groups of the two studies. Klain et al. [45] surveyed groups such as farmers from Costa Rica, tourists in Costa Rica and people from New England, while high school students and university students were the target group of this study. On the one hand, it can be assumed that there is a cultural difference between people from Central and North America and people from Germany, on the other hand, the areas surveyed by Klain et al. [45] are areas where nature conservation (and therefore also RVs) play an important role. Another potential influencing factor could be the large age difference between the groups in the two studies. In this study, rather young persons were surveyed (age_mean_ = 20.26 & 17.51), while the three groups of Klain et al. [45] showed higher average age values (age_mean_ = 32 & 45 & 58). Although it has not yet been empirically analyzed how the RVs relate to age, an age effect as with other environmental variables [19, 73, 83–89] could be possible. A similar result was found in the weighting of items between the studies. Both in the study by Klain et al. [45] and in our study the items RV_other and RV_resp were rated particularly highly. These two items express the responsibility for the environment in relation to other people. The results show that the respondents consider this relationship to be particularly important.

### Group comparison—gender

Females show a significantly higher RV score than males, even if the effect according to the common interpretation [90] is small (d = .273). It is reported that females consider environmental aspects to be more important [91]. Zhang et al. [69] discovered that females are more connected to nature and appreciate the beauty of nature more. Females are more concerned about environmental problems and report to take voluntary behavior to address these problems more often [66]. In addition, females are more engaged in environmental issues and have a more environmental stance [92]. A number of studies showed that females usually have stronger environmental attitudes than males [62, 67, 93, 94] and a cross-national examination of 22 countries found that in 14 countries females showed significantly higher environmental behavior than males [95]. Therefore, gender is a strong predictor for environmental behavior and attitude and females report stronger environmental attitudes and behavior [60]. Vicente-Molina et al. [96] go one-step further and declare that being a male is a factor that decreases the probability of high environmental behavior. For other cultural areas the results are similar but not as clear. Some studies in China report that females show more environmental behavior [97], attitudes and concerns [98] while others did not find a significant gender difference [99]. Research on secondary school students in Nigeria revealed that males tend to show more environmental unfriendly behavior and attitudes than females [100].

The comparison of RVs of males and females reflects these results. Because RVs include stewardship, community, identity, responsibility, care and kinship [45, 47, 51], a higher score for females was expected. Our findings confirm this results and because RVs have the potential to motivate people to pro-environmental actions [57], we add further explanation why females are often more protective towards nature and are more likely to show pro-environmental behavior. The gender difference of RVs could also be explained with the cultural norms and expectations of our western society. The RVs are socially shared as a part of the human habitus in a social group [101]. While males are socialized to be more autonomous and competitive, females are socialized to be more protective, caring and charitable [63]. These characteristics attributed to women because of gender roles are part of RVs but not the connection to nature.

However, this gender difference does not apply to all cultures. Doung & van den Born (70), for example, were unable to detect any difference between the genders in a study of RVs in Vietnam. In Vietnam, this type of value is more important and can be seen as the main stream image of human nature relationship [70]. It can therefore be assumed that gender as a factor has only a minor influence on the characteristics of the RVs due to the high importance in Vietnamese society. Also in a study in Singapore it was found that gender is not a good predictor for a person's RV score [48].

For both connection to nature scales (INS & CNS) our analysis shows no significant difference between the genders for our sampling group. Although connection to nature is a frequently used construct, there are only few studies comparing the gender difference. When developing the CNS Mayer & Frantz [13] did not find a difference between males and females. Also Di Fabio & Rosen [68] only found a negligible effect of gender on connection to nature (d = .05). Our results confirm these findings. One possible explanation why there is a gender difference in the RVs but not in the connection to nature (INS & CNS) could be that human-nature connections are influenced by social institutions. While female gender roles seem to have a positive influence in RVs (such as care or community), these gender roles play no or only a minor role in the connection to nature. In this context, there seems to be an aspect of the human nature connection that affects the RVs alone, but not connection to nature. In Western culture or German society, it might be possible that social institutions (e.g. educational institutions, voluntary associations, family) promote exactly those aspects that play a role in RVs, particularly among women.

### INS, CNS, and RVs during the study of biology

The results suggest that university education in the field of biology has an impact on connection to nature, whereas RVs remain unaffected. Both INS and CNS are significantly higher for university students (first year students and advanced students). The students among themselves show no difference in INS scores and in case of the CNS only a small shift (d = .188).

Current research identifies two main factors responsible for an increase of connection to nature: time spent in nature and environmental education. Various studies reveal a positive link between time spent in nature and connection to nature [6, 8, 13, 52, 55, 83]. The other important influential factor is environmental education, which includes accumulation of knowledge. Education about nature can help to increase the connection to nature (e.g., [21, 22, 58]). Both factors that have a positive influence on the connection to nature are an essential part of the biology studies at Goethe-University Frankfurt. One of the aims of biological education is to convey knowledge about biology and its different disciplines. During their university education, students learn a lot about biological topics, including environmental concerns and environmental knowledge. Another part of the university education is the practical work and acquisition of experience, which the students acquire or build up during outdoor activities in nature. Students have to go on several field trips to special habitats to examine plants and animals. Every student has to create a herbarium that requires countless hours spent in nature. Advanced students also have to do a one-week field trip in which most of the time includes spending time in nature. With this information, it is not surprising that university students show a significantly higher connection to nature than high school students.

However, it is noticeable that first year students do not differ significantly from high school students when measuring the CNS. Only advanced students show a significantly higher CNS score. The reason for this could be the orientation of the measurement instruments. The CNS was developed to measure the emotional attachment to nature [13], while the INS focuses on the cognitive connection [52]. It is possible that a deeper emotional connection is established through intensive environmental education, so that an effect only becomes apparent in advanced students. Cognitive connection to nature, on the other hand, seems to be easier to increase, so that the effect is already visible in first year students.

However, a potential age effect must not be overlooked in the analysis: Older people tend to engage more with nature and try to conserve resources and nature more [84], younger age cohorts usually show higher environmental concerns and a change in behavior is easier to accomplish [85]. A comparison of the results of different studies shows that younger students have a higher preference towards nature conservation [19] than older ones [86, 87]. A similar result can be determined for connection to nature. Older students show lower INS scores [73, 83] than younger pupils [19, 88]. Especially with the beginning of puberty there is a strong cut in the connection to nature. Thus the connection to nature decreases significantly during this time, but increases again afterwards [89]. Both university students and high school students in our sample differed only slightly in age and it can be assumed that both groups were after this puberty bend. Although an age effect cannot be completely ruled out, our results therefore indicate that the higher degree of connection to nature among biology students is due to the study of biology.

In contrast to connection to nature, RVs did not differ significantly between the three tested groups. RVs are the “view on personal and collective well-being” or the knowledge of “what is right” and “habits conducive to a good life […]” [35]. As a part of the human habitus that is social shared [101], these are values that are most likely part of our personality and social identity, which probably makes them more difficult to influence as connection to nature. The constancy of values in itself [102] also supports this interpretation. In biology studies, there is a lack of factors that can change RVs permanently. Such factors that strengthen the relationship to nature and RVs could be, among others, concrete restoration or conservation activities. But also activities in nature with a peer group or a mentor who passes on experiences and passion about nature [35]. Born et al. [57] recommend on-site programs to increase RVs. Uehara et al. [46] were able to demonstrate for an environmental education program among students in the Hinase District in Japan that the RV scores of the participants could be increased. They focused on three factors: a) restoration of the ecosystem, b) active experience of local culture, and c) learning through first hand experience through stories told by people from the local community. These actions could strengthen the connection between people and between people and nature. Even if it is not the main objective of a scientific biology education, such activities could additionally contribute to strengthening the relationship between humans and nature, which in turn would lead to more sustainable behavior and interaction with nature [57]. These programs would be especially desirable for future teachers who were also biology students, as they will later pass on their experiences (as mentors) to their students.

Nevertheless, RVs should be seen as an opportunity for environmental education to encourage people to adopt a more sustainable approach to nature. Especially local environmental education programs should try to promote RVs in order to achieve a more sustainable treatment of land and nature. The request to integrate environmental education programs into the local natural environment and local societies in order to create a connection with the environment and nature is not new. Local projects have the potential to create a link with the environment and to give the content of the program a special meaning for the participants. In this local context, it is particularly possible to have a powerful positive influence on environmental knowledge, environmental values and environmental behavior [16]. RVs also have the opportunity to encourage people and politics to become more steward and increase public participation [103]. Precisely because RVs support pro environmental behavior [57], they could become an important part of environmental education.

### Limitation and further research

Although the study provides an important part of current quantitative RV research, we have to address some limitations. Even our sample size was reasonable, there is a need for additional research on a larger group of people, for example comparing RVs of participants without a natural science background. Studies that aim to observe RVs of different cultural areas could be useful to determine differences and similarities to define common strategies to solve environmental problems. In addition, our age groups are limited: The results only apply to an age group of young adults. Younger students could be more receptive to a change of RVs. Similar results can be found for connection to nature: Younger students tend to show a higher increase of their INS scores compared to older students when attending environmental education programs (e.g. [21, 22]). It is possible that RVs of younger students are less consistent and can be affected by environmental education programs. In order to prove this point, further research is required. Despite our contribution the demand of Schulz & Martin-Ortega [43] for more quantitative research on RVs remains. Especially in the context of environmental education RVs show the potential as a factor to increase pro-environmental behavior and to promote a sustainable use of land and nature. An important methodological limitation is the one-dimensional view of the RVs construct. It can be assumed that RVs consist of multiple factors [45, 47, 51]. Since the aim of this study was a general investigation of the RVs, no subcategories in the RVs were investigated. This would be useful for future studies. Our investigation of the construct is only the beginning of the empirical study on RVs. Further empirical research is required as well as more theoretical framework.

## Conclusion

The study carried out makes an empirical contribution to the investigation of different human-nature connections. It could be shown that there is a gender difference for the concept of the RVs in favor of women. On the one hand, this confirms previous research results that could identify a difference in value between the genders [104, 60, 62, 63], on the other hand, the result provides a further explanation as to why women generally show more environmental behavior [60, 95, 96]. However, the individual human nature connection (measured in this study by INS & CNS) does not show any gender difference, which leads to the conclusion that the individual's own and personal relationship to nature is less subject to social norms and institutional influences (such as gender). In the context of environmental education, it was shown that although the individual human-nature connection can be increased through biological education, the RVs cannot. From this, it can be concluded that a general environmental education, as it is done in biology studies, does not guarantee an increase in RVs. However, since RVs can be an important factor in improving environmental behavior [57], environmental education programs (especially at local level) should try to promote them and integrate them into their programs and curricula. Uehara et al. [46], who were able to demonstrate an increase in RVs through special environmental education, can also provide successful approaches for this.

Although this study makes an empirical contribution to research on human-nature connections, empirical research in the field of RVs is only just beginning and further empirical studies are needed to clarify open questions, such as a comparison of the RVs between different cultures or age groups.

## Supporting information

S1 Fig The used INS-item according to Schultz 2002. The task was "Please choose the illustration that best describes your relationship to nature".
(DOCX)

S1 Table Ratings for each item of the RV-scale, the INS and CNS across gender and education enrollment. (DOC)

S1 Data (XLSX)
