# Supplementary material for: Impact of biological education and gender on students’ connection to nature and relational values
Source: PLoS One. 2020 Nov 5;15(11):e0242004. doi: 10.1371/journal.pone.0242004 (PMC7644009; doi:10.1371/journal.pone.0242004)

**S1 Fig. The used INS-Item according to Schultz 2002. The task was "Please choose the illustration that best describes your relationship to nature."**


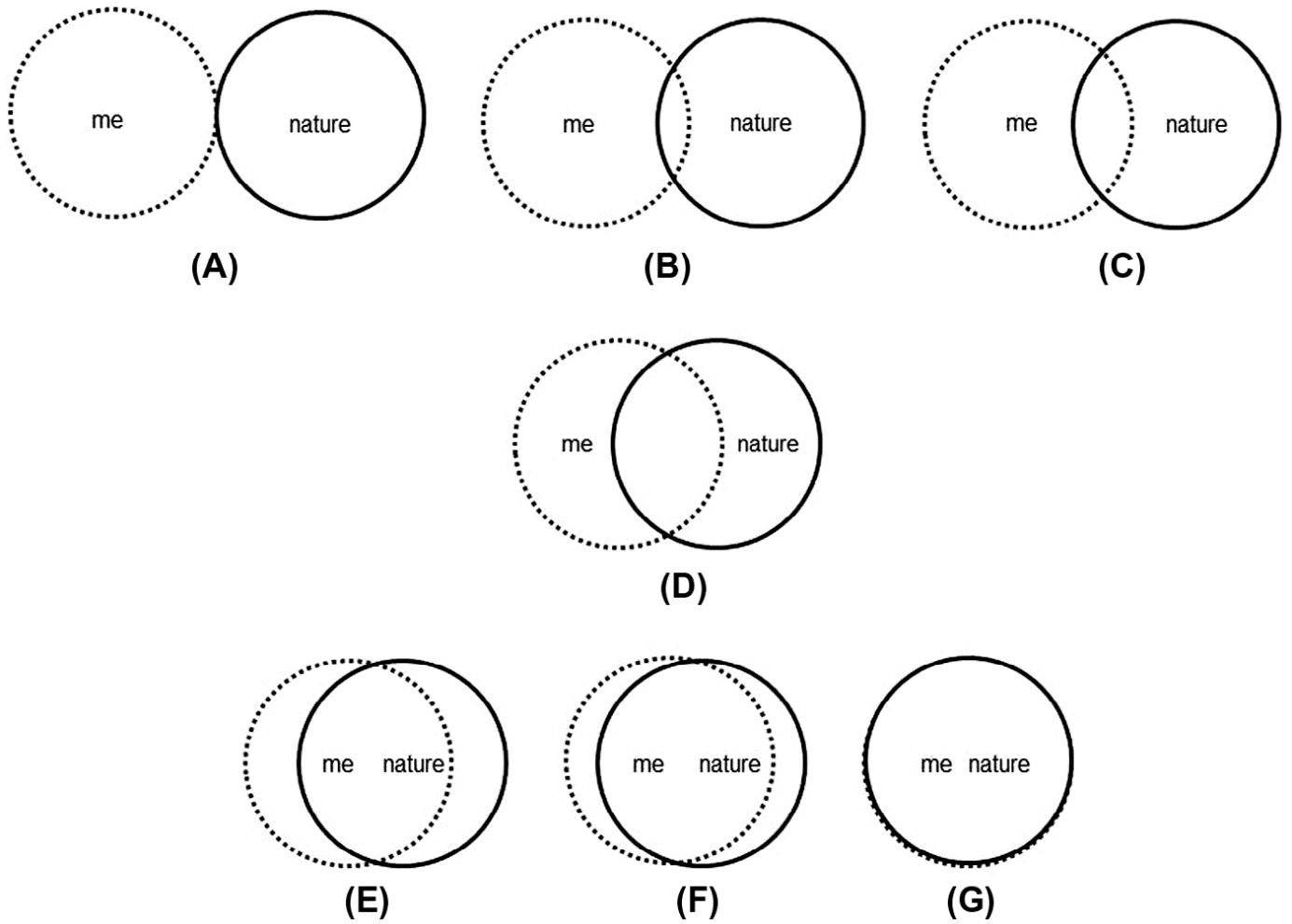

Supplement: S1 Fig — The task was "Please choose the illustration that best describes your relationship to nature". (DOCX) [file pone.0242004.s001.docx]
