# Supplementary material for: Impact of biological education and gender on students’ connection to nature and relational values
Source: PLoS One. 2020 Nov 5;15(11):e0242004. doi: 10.1371/journal.pone.0242004 (PMC7644009; doi:10.1371/journal.pone.0242004)
Supplement: S1 Table — (DOC) [file pone.0242004.s002.doc]

**S1 Table**. Ratings for each item of the RV-scale, the INS and CNS across gender and education enrollment.

| **Mean + S.D.** | **Gender** | | | **education level** | | |
| --- | --- | --- | --- | --- | --- | --- |
|  | **Male** | **Female** | **High school students** | | **First year students** | **Advanced students** |
| **RV** | 3.55 + 0.70 | 3.75 + 0.70 | 3.66 + 0.70 | | 3.67 + 0.72 | 3.74 + 0.69 |
| **RV_iden** | 3.06 + 1.16 | 3.29 + 1.20 | 3.01 + 1.16 | | 3.30 + 1.19 | 3.35 + 1.22 |
| **RV_ resp** | 4.02 + 1.06 | 4.21 + 0.94 | 4.12 + 1.05 | | 4.11 + 0.97 | 4.23 + 0.91 |
| **RV_wild** | 3.25 + 1.28 | 3.41 + 1.28 | 3.37 + 1.30 | | 3.34 + 1.29 | 3.37 + 1.21 |
| **RV_kin** | 2.96 + 1.26 | 3.35 + 1.20 | 3.13 + 1.22 | | 3.25 + 1.25 | 3.28 + 1.22 |
| **RV_health** | 3.71 + 1.15 | 3.91 + 1.03 | 3.85 + 1.06 | | 3.81 + 1.13 | 3.92 + 0.93 |
| **RV_other** | 4.35 + 0.97 | 4.53 + 0.75 | 4.49 + 0.89 | | 4.43 + 0.83 | 4.48 + 0.75 |
| **RV_comm** | 3.50 + 1.24 | 3.55 + 1.20 | 3.62 + 1.20 | | 3.46 + 1.25 | 3.56 + 1.15 |
| **CNS** | 3.02 + 0.90 | 3.07 + 0.81 | 2.96 + 0.82 | | 3.06 + 0.87 | 3.25 + 0.80 |
| **CNS_1** | 2.96 + 1.17 | 3.08 + 1.07 | 2.86 + 1.10 | | 3.10 + 1.14 | 3.24 + 1.03 |
| **CNS_2** | 2.82 + 1.19 | 2.75 + 1.36 | 2.69 + 1.17 | | 2.73 + 1.18 | 3.08 + 1.03 |
| **CNS_3** | 2.78 + 1.12 | 2.82 + 1.05 | 2.66 + 1.07 | | 2.87 + 1.10 | 2.99 + 1.02 |
| **CNS_4** | 3.17 + 1.18 | 3.29 + 1.09 | 3.08 + 1.13 | | 3.31 + 1.17 | 3.45 + 1.02 |
| **CNS_5** | 3.37 + 1.28 | 3.38 + 1.15 | 3.26 + 1.19 | | 3.40 + 1.25 | 3.59 + 1.05 |
| **CNS_6** | 3.04 + 1.31 | 3.08 + 1.19 | 3.18 + 1.25 | | 2.99 + 1.26 | 3.11 + 1.13 |
| **INS** | 4.17 + 1.35 | 4.21 + 1.17 | 3.89 + 1.17 | | 4.37 + 1.29 | 4.33 + 1.10 |
